# Multimodal Imaging of Choroidal Structural in Torpedo Maculopathy

**DOI:** 10.3389/fmed.2023.1085457

**Published:** 2023-02-23

**Authors:** Hao Yuan, Hongliang Dou, Xuemin Li

**Affiliations:** ^1^Department of Ophthalmology, Peking University Third Hospital, Beijing, China; ^2^Beijing Key Laboratory of Restoration of Damaged Ocular Nerve, Beijing, China

**Keywords:** torpedo maculopathy, EDI-OCT, choroid vascularity index, multimodal imaging, classification

## Abstract

**Objective:**

To report a case of torpedo maculopathy with multimodal fundus imaging methods, and apply the choroid vascularity index to quantitatively describe the choroidal structural changes in torpedo maculopathy.

**Case presentation:**

An asymptomatic 41-year-old Chinese woman with an incidentally found yellowish-white macular lesion in her left eye was referred to our hospital. She was unaware of any prior medical conditions. The best corrected visual acuity (BCVA) was 20/20 OD and 20/25 OS, respectively. Fundus exam of her left eye revealed a well-circumscribed torpedo-like hypopigmented lesion in the macula region, and the tapered edge directed toward the fovea. Pigment deposition could be seen in the inferotemporal portion of the torpedo lesion. Fluorescein angiography showed the corresponding window defect without leakage and fundus autofluorescence demonstrated low signal throughout the lesion. Enhanced depth imaging optical coherence tomography revealed outer retinal attenuation, subretinal cavitation, subtle inner choroidal excavation and thinning of outer nuclear layer. The diagnosis of torpedo maculopathy was clinically made. Choroidal vascularity index (CVI) and sub-foveal choroidal thickness (SFCT) were applied to display changes of choroidal structure. The results implied that both subfoveal CVI and SFCT of the affected eye seemed relatively lower when compared with the fellow eye. Optical coherence tomography angiography showed reduced density of the choriocapillaris in the temporal area of the lesion and increased capillary density in the nasal area. Functional examinations, including microperimetry, multifocal electroretinogram and static perimetry also revealed reduced retinal sensitivity, decreased stimulated amplitude and suspected scotoma in the lesion area. After 12 months of follow-up, the patient’s visual acuity and the clinical appearance of the lesion were unchanged.

**Conclusion:**

The torpedo maculopathy may be identified by abnormal appearance with multimodal imaging. Decreased choroidal vascularity in the lesion area measured quantitatively by choroid vascularity index may play a role in pathogenesis of torpedo maculopathy.

## Introduction

Torpedo maculopathy (TM), first described as a ‘hypopigmented nevus of the RPE in the macula’ by Roseman and Gass in 1992, is generally regarded as a congenital developmental abnormality of retinal pigment epithelium (RPE) with typical morphology (1). It is characterized by an asymptomatic, unilateral, well-circumscribed, hypopigmented macular lesion located along the horizontal raphe (2). The lesion appears as a torpedo or oval defect in the retinal pigmented epithelium with tapered edges directed toward the fovea (3). According to the optical coherence tomography (OCT) features, Wong et al. classified TM into two types: Type 1 TM demonstrates outer retinal attenuation without retinal cavitation; Type 2 TM is characterized by outer retinal attenuation and outer retinal cavitation which may or may not be associated with inner choroidal excavation (4). The thinning and irregularity of outer retina and the disruption of RPE-Bruch complex are the morphological characteristics (4).

Despite the unique characteristic appearance of torpedo maculopathy, its etiology is still unknow and controversial. It has been hypothesized to be the result of a number of possible aberrant processes, including malformation of choroidal capillary layer (2), incomplete arcuate bundle differentiation (5, 6) or defect of RPE development at the temporal fetal bulge (7). Among the several suspected causes, the abnormality of choroid vessels is recently considered as an essential process in the pathogenesis, which has been preliminarily visualized by OCT angiography (OCTA). Ali et al. demonstrated the OCTA findings among TM patients, including the absence of RPE and choroidal capillaries in the lesions, the preservation of retinal superficial capillary plexus and decrease of deep capillary plexus (8). However, there is still lack of quantitative and clear assessment of choroidal structural changes in torpedo maculopathy.

The application of enhanced depth imaging (EDI) technique in OCT has allowed non-invasive, quantitative assessment of the choroid (9). Choroidal thickness has been regarded as the main surrogate marker for describing choroidal structural changes in majority of studies concerning chorioretinopathy (8, 10, 11). However, considering the heterogeneous nature of choroid, it was unclear whether the vascular and stromal components were similarly or differentially affected in patients. Recently, choroidal vascularity index (CVI), describing the proportion of vascular area in the choroid based on EDI-OCT images, was proposed as a novel tool to quantitatively assess the choroidal structural changes (12). Because CVI encompasses changes in both the vascular and stromal component of the choroid, it gives unique and additional structural information compared to the choroidal thickness and has been widely utilized (12–14).

We present a case of torpedo maculopathy with multimodal fundus imaging methods, and introduce the choroid vascularity index to quantitatively describe the choroidal structural changes in torpedo maculopathy.

## Patient information

An asymptomatic 41-year-old Chinese woman with an incidentally found yellowish-white macular lesion in her left eye during a routine examination was referred to consult the retinal specialist for further assessment. She denied any family history of inherited retinal disease, history of ocular trauma, exposure to retinal toxic drugs, infectious choroidal or retinal diseases such as toxoplasmosis. No relevant interventions were conducted.

## Clinical findings

The patient received complete ophthalmic examination and multimodal fundus imaging, including fundus photography, infra-red and fundus autofluorescence (FAF) imaging, raster scanning of enhanced depth imaging spectral-domain optical coherence tomography (EDI SD-OCT; B-scan; Spectralis; Heidelberg, Germany) and OCT angiography (Optovue, Fremont, CA), multifocal electroretinogram (RETI Scan, ROLAND CONSULT, Germany) and microperimetry (microperimeter-1, Nidek, Italy).

**Figure 1:**
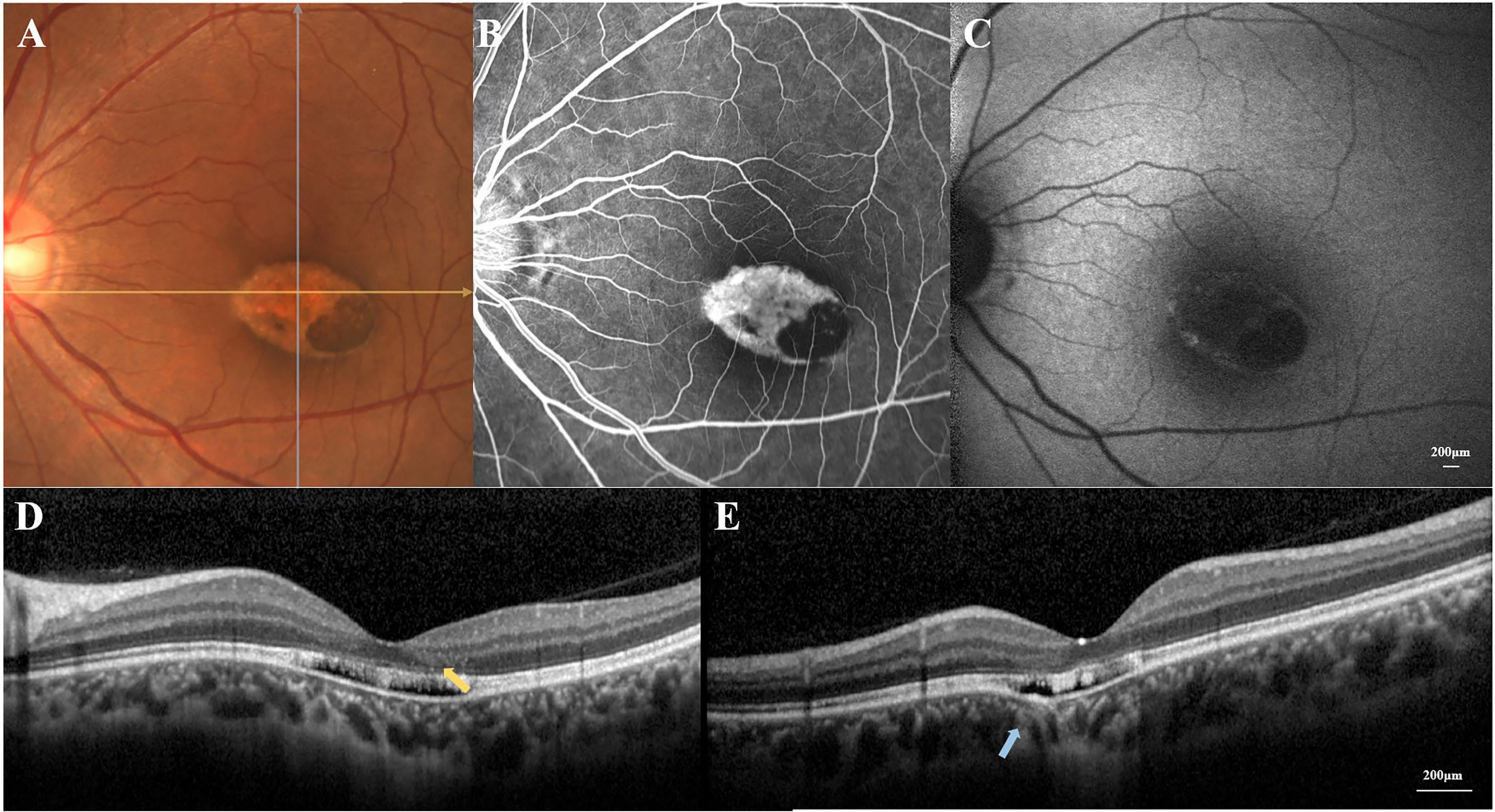
**(A)** Color fundus photograph revealed a well-circumscribed torpedo-like hypopigmented macula lesion. **(B)** Fluorescein angiography showed a corresponding window defect without leakage. **(C)** Fundus autofluorescence demonstrated loss of autofluorescence throughout the lesion with some hyper-autofluorescence signals along the margin. **(D)** The horizontal OCT scan revealed the disruption of the external retinal, the mild outer retinal cavitation, and the thinning of outer nuclear layer (yellow arrow). **(E)** The vertical OCT scan showed subtle inner choroidal excavation (blue arrow).

With the refraction of −5.50DS/−0.50 DC × 42° OD and −2.50DS/−0.50 DC × 150° OS, she had best corrected visual acuity of 20/20 OD and 20/25 OS. The intraocular pressure measured by non-contact tonometer was 15.7 mmHg (15) and 16.3 mmHg (OS). The bilateral anterior segment examinations revealed no obvious abnormalities. A well-circumscribed torpedo-like hypopigmented lesion was found in the macula region of the left eye, and the tapered edge was oriented toward the fovea (Figure 1A). Pigment deposition could be seen in the inferotemporal portion of the torpedo lesion. A corresponding window defect without leakage was observed on fluorescein angiography, with slight variegated fluorescence at the nasal lesion (Figure 1B). Low autofluorescence throughout the lesion and some hyper-autofluorescence spots along the margin of lesion were observed (Figure 1C).

**Figure 2:**
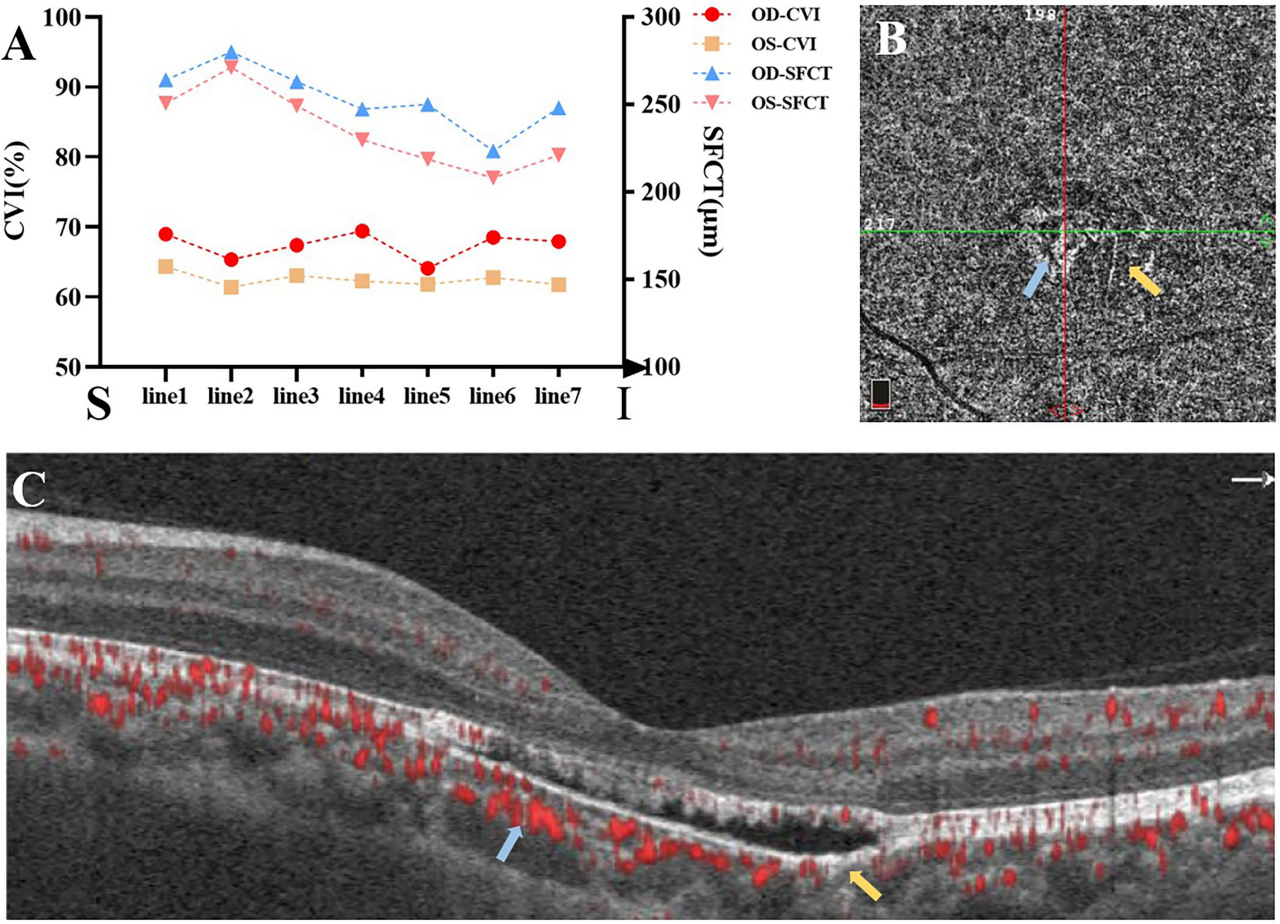
**(A)** The choroidal vascularity index (CVI) and sub-foveal choroidal thickness (SFCT) of seven horizontal lines from superior to inferior passing through the fovea (S: superior; I: inferior). **(B,C)** OCTA showed reduced density of the choriocapillaris in the temporal lesion (yellow arrows) and increased density in the nasal lesion (blue arrows).

Choroidal vascularity index (CVI) and sub-foveal choroidal thickness (SFCT) were calculated by methods as previously described (for details in Supplementary material; 12, 13). The results implied that both subfoveal CVI and SFCT of left eye seemed relatively lower when compared with right eye (Figure 2A).

**Figure 3:**
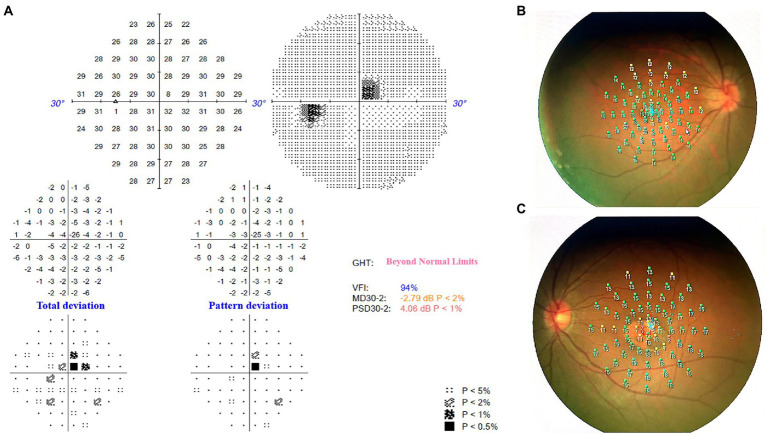
**(A)** Humphrey static perimetry implied the comparative scotoma in the superior-nasal central visual field of left eye, which was consistent with the location of the macula lesion. **(B)** Microperimetry revealed normal sensitivity of macula fovea in the right eye. **(C)** Microperimetry revealed the reduced sensitivity of macula fovea in the lesion area of left eye.

**Figure 4:**
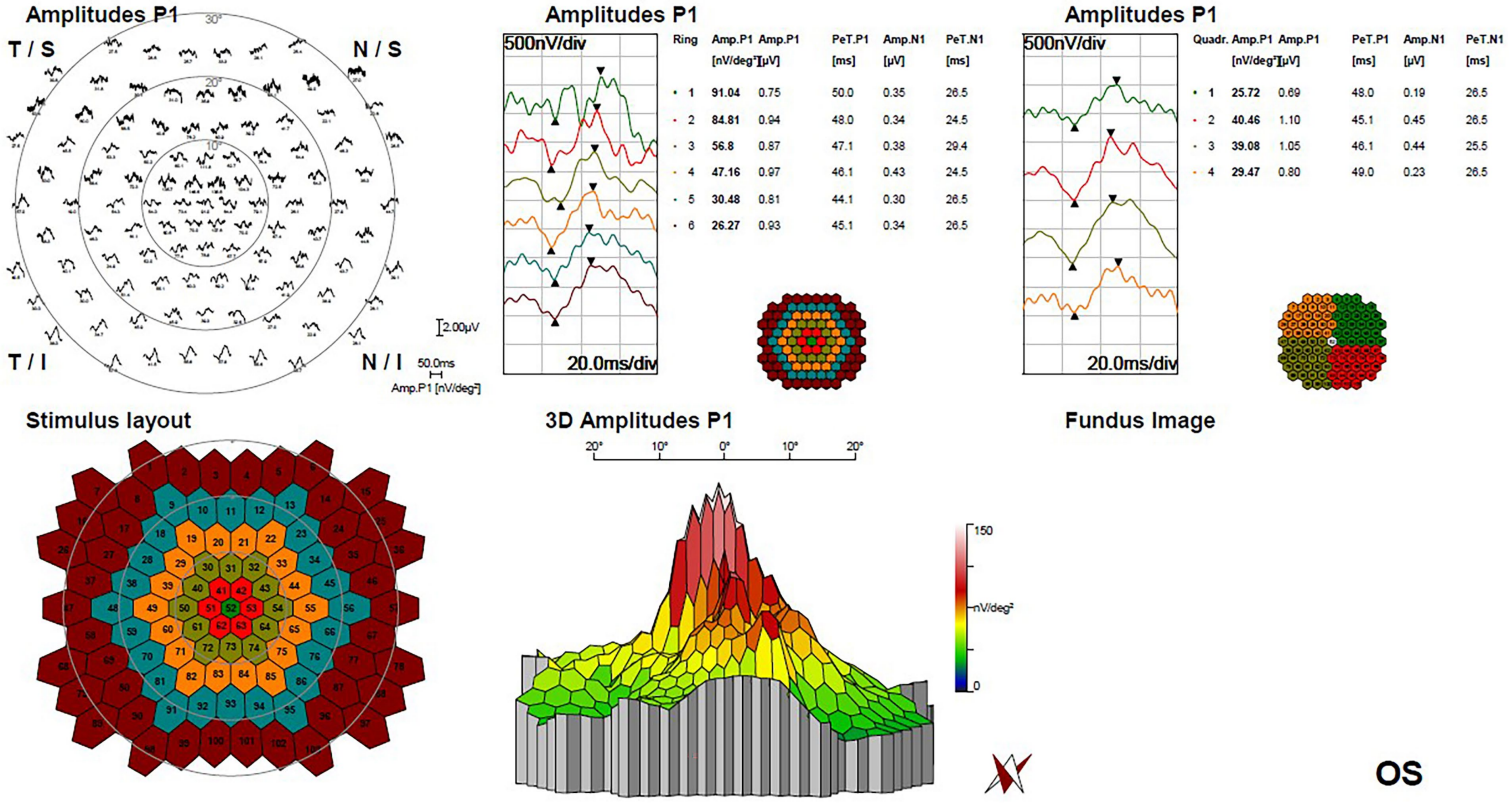
Multifocal electroretinogram of left eye demonstrated a significant focal amplitude decrease, especially in the temporal portion of macula lesion.

The horizontal OCT scan through the lesion revealed typical changes, including the disruption of the myoid, ellipsoid and interdigitation zones, the irregularity and attenuation of RPE-Bruch complex, the mild outer retinal cavitation, and the thinning of outer nuclear layer. These morphological abnormalities of retinal out layers exhibited obviously in the temporal fovea (Figure 1D). Additionally, the vertical scan showed subtle inner choroidal excavation in the inferior portion of the lesion corresponding to the pigmented area and meanwhile, mild increased signal transmission in the choroid (Figure 1E). The inner retinal layers appeared normal and well organized. Based on the classification mentioned above, the macula lesion in this case could be categorized as Type 2 TM. OCTA showed reduced density of the choriocapillaris in the temporal area (pigmented area) of the lesion and increased density in the nasal area (hypopigmented area; Figures 2B,C). Microperimetry (Figures 3B,C) precisely revealed the reduced sensitivity of macula fovea and multifocal electroretinogram (Figure 4) demonstrated a significant focal amplitude decrease, especially in the hyperpigmented area, which was consistent with the result of Humphrey static perimetry that revealed the suspected scotoma in the superior-nasal central visual field (Figure 3A).

Based on the results of multimodal fundus images, a diagnosis of torpedo maculopathy was made. Considering the differential diagnosis, congenital lesions of RPE (e.g., congenital hypertrophy of RPE, Gardner syndrome) were excluded due to the lack of RPE thickening, and inactive chorioretinal scars (e.g., posterior uveitis, toxoplasmosis) were ruled out because of the absence of typical inflammation scar and serious retinal disorganization that are often occurred in acquired chorioretinal lesions. Given the lack of symptoms and the absence of neovascular activity, the patient was followed up per annual without any interventions. One year later, the patient’s visual acuity and the clinical appearance of the lesion were unchanged when she was checked at our eye center.

## Discussion

We present a case of torpedo maculopathy with multimodal fundus imaging methods. To our knowledge, the choroid vascularity index was applied to quantitatively describe the choroidal structural changes in TM for the first time and found decreased choroidal vascularity in the lesion area. Up to now, the etiology of torpedo maculopathy still remains unclear. It has been hypothesized to be the result of a number of possible aberrant processes, including malformation of choroidal capillary layer, incomplete arcuate bundle differentiation (5) or defect in RPE development at the temporal fetal bulge (7). However, the abnormal vasculature development of choroid has always been regarded as an essential process in the pathogenesis of TM, which was preliminarily been demonstrated by OCTA (8). Whereas, there is still lack of quantitative and systematic assessment for choroidal structural changes in torpedo maculopathy. Recently, the application of enhanced depth imaging in optical coherence tomography has enabled comparatively clear visualization of choroid structure and quantitative assessment of choroidal vascular density (9). Rohl et al. adopted EDI-OCT to explore the characteristics of choroid in a TM patient and did not found any abnormality in choroidal thickness beneath the torpedo lesion (16), implying that choroidal thickness might not directly correlate with the presence of torpedo maculopathy. Moreover, considering the heterogeneous nature of choroid and the impact of confounding factors including age, blood pressure, axial length and intraocular pressure, choroid thickness might not be an appropriate index to evaluate the choroid structure in TM (17).

Choroidal vascularity index, calculating the proportion of vascular area in the choroid based on EDI-OCT images, was proved to be independent from systemic and ocular factors and proposed as a novel means to quantitatively assess both the vascular and stromal component of the choroid, which could give unique and additional structural information compared to the choroidal thickness. It has been widely utilized in the evaluation of choroid structure (12–14). Lower CVI with similar choroidal thickness has been reported in many other macula diseases (18–20). We firstly applied choroidal vascularity index to evaluate choroid structure components in this patient of TM. The results revealed that, comparing with normal fellow eye, the subfoveal CVI and SFCT of affected eye relatively decreased. Reduced choroidal vascularity and choroidal excavation in TM lesion may be the results of a more marked atrophy of the vascular component instead of stroma. Ali et al. also found the reduction of choroidal capillary density measured by OCTA in TM lesions, which was similar to our result. The reduction of vascular component might be mainly located in the capillary layer (8). However, in another early-stage TM case with same ethnicity, Ding et al. reported that OCTA of choroid capillary layer revealed increased density of the choroidal vasculature corresponding to the area of the lesion, and the superficial and deep layers appeared normal (21). Therefore, the alterations of choroidal vascularity in TM might be inconsistent at different periods of diseases.

Low amplitude in ERG, decline of macula sensitivity by microperimetry and comparative scotoma by static visual field test of the affected eye were found in this case. Several previous studies also reported the decreased focal amplitude in multifocal ERG of TM patients (22–24), and Kumar et al. even found subnormal response in both eyes with slightly greater reduction in the affected eye corresponding to the Torpedo lesion (24). Similarly, in our case, microperimetry sensitivity also showed low values even in the fellow eye, which suggested the potential subclinical visual impairment of the normal fellow eye among TM patients. The possible explanation of visual function impairment is that focal choroid capillary atrophy compromise metabolites and oxygen supply for the RPE and photoreceptors, leading to the degeneration of retinal outer layer. And the abnormality of cone photoreceptor and RPE in TM have been detected by adaptive optics-assisted fundus imaging (25–27). Hugo et al. found that within the TM lesion, the cone density was lower and the spacing between cones was higher (26). Vienola et al. revealed the alterations of RPE density and morphology, even extending beyond the bounds of the clinically defined lesion (27). Therefore, we speculated that the degree of choroid vascularity atrophy might be closely associated with the retinal sensitivity and visual function in TM, and CVI might potentially enable the acquisition of prognostic data to monitor the progression of disease and predict the visual function.

On the other hand, our result also suggested that, apart from choroid thickness, CVI appeared to be another sensitive biomarker in detecting choroidal changes among TM patients. However, due to the rarity of TM, we could only assess choroid structure at the individual level. To determine whether this association will be clinically relevant or not, the data need to be validated with a large scale longitudinal prospective study. Despite that the reconstruction of choroidal structure and vascularity in TM has been detected, the underlying cause of these changes in the choriocapillaris remains unknown. The choroidal angiopathy could either be part of the primary pathogenesis of torpedo maculopathy or a consequence of RPE malformation.

Generally, TM was regarded as a hypopigmented macula lesion (2), while variably hyperpigmented portion had been described in a few cases (2, 16, 28). In our case, it is noteworthy that the lesion could be divided into hypopigmented area and hyperpigmented area, and the two portions showed distinctly different pathological characteristics. The hyperpigmented area exhibited more atrophic structure changes, including reduced choriocapillaris density, remarkable thinning of outer nuclear layer and subtle inner choroidal excavation, which were not presented in the hypopigmented area. The functional examinations also revealed reduced macular sensitivity, decreased stimulated amplitude and comparative scotoma in the hyperpigmented area, which confirmed an abnormal functional component in the local hyperpigmented portion and was consistent with the structural changes. Other investigations also displayed pigmented TM tending to be associated with the outer retina thinning, choroidal excavation, retinal sensitivity decrease and progressive morphological changes over time (2, 16, 29), and one patient finally developed choroid neovascularization in the focal hyperpigmented area after 5-year follow-up (29). The hyperpigmented portion in TM lesion, comparing with the classical hypopigmented area, may be more likely to link with atrophic retinal and choroidal degeneration and visual functional impairment. The existence and proportion of hyperpigmented area in the TM lesion might be used to evaluate the severity and progression of disease, which needs to be validated with a large scale longitudinal prospective study to confirm its correlation with the structural changes and function prognosis of TM. This patient presented asymptomatic, but mild decline of best corrected visual acuity, macula sensitivity decrease and low ERG amplitude were detected in left eye with TM. Possible reason is the TM lesion had involved fovea. The majority of previous studies demonstrated that TM lesion located in the temporal macula (2, 3, 6, 25). Therefore, apart from the characteristics of lesion appearance, the location of lesion in macula might also play an essential role in visual function of TM.

There are several limitations in this study. Choroid vascularity index was measured by a series of B-scans across the fovea. We might obtain more information if the same technique was applied to a volume scan in broader area of the macula. Besides, long-term cohort studies would be needed to determine choroid structure change in TM over time, and validate the repeatability and reliability of CVI as a reliable biomarker.

## Conclusion

This case provided multimodal imaging findings in torpedo maculopathy. Reduced choroidal vascularity in the lesion area measured quantitatively by choroid vascularity index may play a role in pathogenesis of torpedo maculopathy.

## Data availability statement

The raw data supporting the conclusions of this article will be made available by the authors, without undue reservation.

## Ethics statement

The studies involving human participants were reviewed and approved by the Ethics Committee of Peking University Third Hospital. The ethics committee waived the requirement of written informed consent for participation. This report does not contain any personal information that could lead to the identification of the patient. Therefore, the consent to publish the case report was not obtained.

## Author contributions

All authors listed have made a substantial, direct, and intellectual contribution to the work and approved it for publication.

## Funding

This project was supported by Beijing Municipal Natural Science Foundation (7202229) from Beijing Municipal Science and Technology Commission.

## Conflict of interest

The authors declare that the research was conducted in the absence of any commercial or financial relationships that could be construed as a potential conflict of interest.

## Publisher’s note

All claims expressed in this article are solely those of the authors and do not necessarily represent those of their affiliated organizations, or those of the publisher, the editors and the reviewers. Any product that may be evaluated in this article, or claim that may be made by its manufacturer, is not guaranteed or endorsed by the publisher.
