# Supplementary material for: Multimodal Imaging of Choroidal Structural in Torpedo Maculopathy
Source: Front Med (Lausanne). 2023 Feb 23;10:1085457. doi: 10.3389/fmed.2023.1085457 (PMC9996047; doi:10.3389/fmed.2023.1085457)
Supplement: Supplementary file 1 [file Data_Sheet_1.pdf]

The detailed calculation methods of choroidal vascularity index (CVI) and sub-foveal choroidal thickness (SFCT): The macular region was scanned using a raster scanning mode with seven horizontal lines (30°×7.5°) centered on the fovea. Each B-scan was 6.3 mm in length and spaced 260μm apart from each other. The entire length of seven horizontal OCT scans passing through the fovea was selected for analysis, named as line1-7 from superior to inferior, respectively. The images acquired were processed on ImageJ software (version 1.53; provided in the public domain by the National Institutes of Health, Bethesda, MD, USA; http://imagej.nih.gov/ij/) for further analysis. Firstly, the OCT scans were transferred to 8-bit images by ImageJ default setting. The application of Niblack's auto local threshold tool allowed demarcation of choroidal luminal area (LA) and stromal area (SA). Then, with the polygon tool, the total choroidal area (TCA) was selected by manual plotting of the upper border marked at the RPE and the lower border marked at the choroid-sclera junction. To allow computation of size of LA, the image was then converted back to an RGB image by the color threshold tool. Finally, CVI was calculated as the ratio of LA to TCA. The sub-foveal CVIs of seven scans were calculated from superior to inferior, respectively. SFCT was taken as the distance between the Bruch membrane (located at the lower edge of the RPE) and the choroid-scleral interface, which was then measured using the in-built software calipers tool at the fovea.
